# Synthesis of Organic Matter in Aqueous Environments Simulating Small Bodies in the Solar System and the Effects of Minerals on Amino Acid Formation

**DOI:** 10.3390/life11010032

**Published:** 2021-01-06

**Authors:** Walaa Elmasry, Yoko Kebukawa, Kensei Kobayashi

**Affiliations:** Department of Chemistry and Life Science, Graduate School of Engineering Science, Yokohama National University, 79-5 Tokiwadai, Hodogaya-ku, Yokohama 240-8501, Japan; walaa-elmasry-hm@ynu.jp (W.E.); kobayashi-kensei-wv@ynu.ac.jp (K.K.)

**Keywords:** formaldehyde, ammonia, amino acids, olivine, phyllosilicates, small Solar System bodies, aqueous alteration

## Abstract

The extraterrestrial delivery of organics to primitive Earth has been supported by many laboratory and space experiments. Minerals played an important role in the evolution of meteoritic organic matter. In this study, we simulated aqueous alteration in small bodies by using a solution mixture of H_2_CO and NH_3_ in the presence of water at 150 °C under different heating durations, which produced amino acids after acid hydrolysis. Moreover, minerals were added to the previous mixture to examine their catalyzing/inhibiting impact on amino acid formation. Without minerals, glycine was the dominant amino acid obtained at 1 d of the heating experiment, while alanine and β-alanine increased significantly and became dominant after 3 to 7 d. Minerals enhanced the yield of amino acids at short heating duration (1 d); however, they induced their decomposition at longer heating duration (7 d). Additionally, montmorillonite enhanced amino acid production at 1 d, while olivine and serpentine enhanced production at 3 d. Molecular weight distribution in the whole of the products obtained by gel chromatography showed that minerals enhanced both decomposition and combination of molecules. Our results indicate that minerals affected the formation of amino acids in aqueous environments in small Solar System bodies and that the amino acids could have different response behaviors according to different minerals.

## 1. Introduction

Meteorites and interplanetary dust particles which came from small bodies in our Solar System, including comets and asteroids, provide valuable data on the chemical processes that occurred in the early Solar System. Extraterrestrial delivery of organic compounds including amino acids to the early Earth prior to the generation of life may have been important for the origin of life. Some carbonaceous chondrites, which include the most primitive meteorites, are mostly made up of silicates but contain abundant organic carbon up to 3–5 wt.% [1,2,3], which exists as insoluble organic matter (IOM) and soluble organic matter (SOM). Chemical records of the prebiotic synthesis reactions that occurred before and during the formation of the early Solar System and during subsequent alteration of the parent bodies are mostly provided by the SOM in carbonaceous chondrites. As some of these compounds could be precursors to biological molecules [4,5,6], the origin of these organic compounds is of interest in understanding the origin of life on Earth. In the soluble fraction of meteorites, carboxylic acids at approximately 300 parts per million (ppm) are the most abundant class of compounds, followed by more than eighty different amino acids at approximately 60 ppm total in the Murchison CM2 carbonaceous chondrite [5]. The early Solar System contained a wide array of organic compounds that were abiotically formed, including interstellar organics, that were incorporated into planetesimals and the parent bodies of meteorites and then finally delivered to the early Earth. By proton irradiation of a gas mixture of CO, NH_3_, and H_2_O, complex organic compounds could be synthesized, simulating the interstellar complex organic compounds that are known to produce amino acids after acid hydrolysis [7,8,9]. Also, a wide variety of complex organic compounds, including amino acids, can be formed through UV irradiation of simple interstellar ice mixtures and warming up prior to the accretion of planetary bodies [10,11,12,13,14].

Hydrothermal reactions affect both organic matter [15,16,17] and mineralogical composition [18,19]. Most of the chondrite parent bodies have experienced aqueous alteration soon after their accretion [18], since enough internal heating has been produced through the radioactive components (mostly ^26^Al) [20,21] to melt ice. These aqueously altered meteorites contain up to 1.3 wt.% H in water/OH mainly in the structure of phyllosilicates [22]. The continuous interaction of water with minerals indicates progressive aqueous alteration to various degrees in carbonaceous chondrites (e.g., [18,23,24,25,26]). For example, the presence of hydrous minerals such as serpentine, which is the alteration product of olivine, indicates that aqueous alteration has occurred in the parent bodies of carbonaceous chondrites [18].

Chondritic insoluble organic matter was proposed to be formed through the reaction of interstellar formaldehyde followed by condensation and carbonization, possibly during a hydrothermal alteration in small bodies of the Solar System [27]. Furthermore, Kebukawa et al. [28,29] illustrated that the presence of ammonia significantly enhances the yield of solid organic matter from aldehydes and simultaneously produced amino acids. Various organic compounds could be also produced from formaldehyde and ammonia generated from hexamethylenetetramine degradation in environments simulating hydrothermal conditions of asteroids at 150 °C and alkaline pH for 31 days [15]. Therefore, organic compounds, including amino acids, may have been synthesized from aldehydes and ammonia in aqueous environments in small bodies. Minerals could affect hydrothermal reactions of organic matter. Rotelli et al. [30] reported the catalytic effects of minerals of carbonaceous chondrites in the presence of water and formamide in the production of a diverse range of organic compounds (nucleobases, amino acids, and carboxylic acids) that are important for the origin of life and confirmed that some reactive minerals could have served as catalysts to promote increasing organic complexity in chemical evolution. It is suggested that phyllosilicates may have acted as absorbents and catalysts in the early Solar System for the organic reactions [31] since the key mechanisms involved are their surface reactions and interlayer space reactivity, which can explain their function as absorbent or catalyst [32,33]. The presence of phyllosilicates could catalyze the organic compounds to be more abundant and more complex, and the nature of the phyllosilicate impacted the abundance and the molecular composition of the final organic compounds, including amino acids [34,35]. The different chemical composition of the phyllosilicates suggests different oxidation states, as well as the release of different cations in solution by dissolution, possibly leading to the formation of various complexes rich in organic matter, and, in turn, different catalyzing/inhibiting reactions [34]. Yamashita and Naraoka [36] demonstrated that the presence of olivine powder promoted the formation of alkylpyridines from aldehydes and ammonia (the Chichibabin synthesis). Overall, the presence and the nature of the silicates could affect the abundance and molecular composition of the final organic compounds in primitive bodies during hydrothermal alteration. In the current research, we evaluated the effect of minerals, namely, olivine, montmorillonite, and serpentine for amino acid production in conditions simulating water-bearing parent bodies.

## 2. Methods

### 2.1. Samples and Hydrothermal Experiments

The organic compounds were synthesized using a starting solution (200 μL) containing 77.3 μL of 36.0–38.0% (13 M) formaldehyde aqueous solution, 8.4 μL of 25.0–27.9% (14 M) ammonia aqueous solution, and water (145 μL), at a molar ratio of H_2_CO:NH_3_:H_2_O = 9:1:100 (5 M H_2_CO and 0.6 M NH_3_). We refer to the product as “FAW” after formaldehyde–ammonia–water. The experiments were conducted following the method of the amino acid synthesis experiments of Kebukawa et al. [29], but without glycolaldehyde and Ca(OH)_2_. To evaluate the effect of minerals in chondritic meteorites on amino acid production, we added minerals (10 g/L) to the FAW solution mixture. The minerals used were olivine ((Mg, Fe)_2_SiO_4_) from San Carlos, Arizona, USA, Na-montmorillonite ((Na,Ca)_0.33_(Al, Mg, Fe)_2_Si_4_O_10_(OH)_2_·*n*H_2_O)—“SWy-1” from the Clay Minerals Society, and serpentine (antigorite) ((Mg,Fe)_3_Si_2_O_5_(OH)_4_) from Miyatsu, Kyoto, Japan, purchased from Nichika Corp. #14-4-12-1. The mineral powders were washed with hexane before use, and then fully dried at room temperature (RT). Each solution was flame-sealed into glass tubes after O_2_ elimination from the system using a vacuum after cooling with liquid nitrogen, and then these tubes were heated at 150 °C over reaction periods of 1 d, 3 d, or 7 d in an oven (ETTAS HTO-300S). Each set of conditions was repeated three or more times. Control samples were prepared using a starting solution contained formaldehyde and water (without ammonia) at a molar ratio of H_2_CO:H_2_O = 9:100. Minerals (10 g/L) were added to the solution mixture under the same experimental conditions (150 °C, at 1 d, 3 d, or 7 d). Unheated samples (0 day samples) were also prepared using formaldehyde, ammonia, and water at a molar ratio of H_2_CO:NH_3_:H_2_O = 9:1:100 as a starting solution. In addition, unheated samples with minerals (10 g/L) were prepared. Both control and unheated samples were analyzed with the same procedures. All the glassware used was baked at 500 °C for at least three hours prior to use in all experiments and the Millipore Milli-Q^®^ system was used to purify water used in this synthetic experiment and the subsequent analytical procedures.

### 2.2. High-Performance Liquid Chromatograph (HPLC) Analysis

After heating, an aliquot (150 μL) of the FAW was mixed with 150 μL of 6 M HCl for acid hydrolysis and heated at 110 °C for 24 h to convert them into their corresponding amino acids. Most of the amino acids are stable under the previous classical conditions of hydrolysis and can be calculated quantitatively [37,38]. The FAW was dried by vacuum centrifugation at 60 °C after acid hydrolysis. The hydrolyzed fraction underwent desalting with a cation-exchange resin (AG 50W-X8 resin). The resin (5 mL) was sequentially prewashed with the following liquids: 1 M HCl (20 mL), water (20 mL), 1 M NaOH (20 mL), water (20 mL), 1 M HCl (20 mL), and water (20 mL). After filtration with polytetrafluoroethylene (PTFE) membrane filters (DISMIC-13HP 0.45 μm) and rinsing with 0.1 M HCl (1 mL) and water (20 mL), an aliquot of 0.1 M HCl sample solution (1 mL) was inserted into the column. Then, the amino acids-containing solution was eluted successively with 10% NH_3_ aqueous solution (15 mL) and water (20 mL). The NH_3_ + H_2_O elute was centrifuged dried at 60 °C, and the dried sample was fully dissolved in 1 M NaOH (0.5 mL) and 1 mL of water, then dried again by vacuum centrifugation at 60 °C. Finally, the dried sample was dissolved by adding 0.1 M HCl (40 μL) and 160 μL of water. The control and unheated samples were treated under the same previous preparation protocol (acid hydrolysis followed by desalting).

A 1-μL aliquot of each FAW solution with and without minerals was filtrated through cellulose acetate membrane filters (DISMIC-3CP 0.45 μm) and analyzed by using an Ultra-High-Performance Liquid Chromatograph (UHPLC, Nexera X2, Shimadzu, Kyoto, Japan) system equipped with an autosampler (SIL-30AC). To separate the amino acids, a reversed-phase column (Inertsil-ODS4 column, 100 mm L. × 3.0 mm I.D.) was used. The UHPLC system was equipped with an LC-30AD solvent delivery unit, RF-20Axs fluorescence detector, CBM-20A/lite system controller, and CTO-20AC column oven which maintains the temperature at 35 °C. Chromatography was performed by gradient elution with mobile phases of (A) a solution of 15 mmol/L potassium dihydrogen phosphate and 5 mmol/L dipotassium hydrogen phosphate, and (B) acetonitrile:methanol:water = 45:40:15 (*v*/*v*/*v*) at a flow rate of 0.8 mL/min. Fluorescence detection used excitation at 350 nm and emission at 450 nm. The derivatization reagents used were 0.1 mol/L borate buffer (pH 9.2) solutions of mercaptopropionic acid (MPA) (a mixture of 10 mL borate buffer and 10 μL MPA) and o-phthalaldehyde (OPA) (a mixture of 0.7 mL borate buffer, 10 mg OPA, 0.3 mL ethanol, and 4 mL water), acetonitrile solution of chloroformic acid 9-fluorenylmethyl (FMOC) (a mixture of 25 mL acetonitrile and 10 mg FMOC), and 0.1 mol/L potassium phosphate buffer (a mixture of 0.34 mL 85% phosphoric acid and 0.68 g potassium dihydrogen-phosphate) (pH 2). For peak identification, commercial standard solutions of the amino acid (Wako Amino Acids Mixture Standard Solution, Type B and Type AN-2) were used. In the control experiments (without ammonia under the same experimental conditions), only trace amounts of amino acids were detected after acid hydrolysis. The concentrations (μM) of amino acids are control-corrected values that were obtained by subtraction of the control samples from the amino acid quantities of the FAW. The error bars reflect experimental uncertainties based on the repeated heating experiments under the same protocol.

### 2.3. FTIR Micro-Spectroscopy Analysis

Fourier transform infrared (FTIR) micro-spectroscopy was performed to evaluate the evolution of the molecular structure of the organic compounds derived from the FAW solution that occurred due to the heating period and mineral addition. After hydrothermal experiments, the soluble fraction was separated through centrifugation, and then one droplet was placed on a CaF_2_ plate (13 mm diameter × 1 mm thickness), and dried. IR absorption spectra were obtained through a Fourier-transform infrared micro-spectrometer (micro-FTIR; FT/IR-6100typeA+IRT-5200, JASCO), equipped with a mercury-cadmium-telluride (MCT) detector, a ceramic IR light source, a germanium-coated KBr beam splitter, and ×16 Cassegrain mirrors. The microscope and the FTIR were continually purged with dry N_2_ during analysis. A total of 128 scans of IR transmission spectra were accumulated in the wavenumber range of 8000–400 cm^−1^, with a wavenumber resolution of 4 cm^−1^, with a 20 μm × 20 μm aperture. Background spectra were obtained via blank areas of the CaF_2_ plates adjacent to the samples. Several different points (3–5) were analyzed for each sample, and we calculated the required peak ratios for each spectrum. All the peak ratios were then averaged for each sample.

### 2.4. Gel Filtration Chromatography Analysis

Gel filtration chromatography is a chromatographic technique in which molecules are separated by their molecular weight and was used to evaluate the changes in the molecular weight of FAW samples in response to the heating duration and mineral addition. After the hydrothermal experiments, all FAW samples were filtrated through PTFE membrane filters (DISMIC-13HP 0.45 μm). A Shimadzu HPLC system was used, which was equipped with an SPD-M20A photodiode detector, two LC-20AD pumps, DGU-20A5 degassing unit, and a CTO-20AC column oven that provides precise temperature control at 30 °C. The absorbance detection wavelength was at 260 nm and the sample injection amount was 5 μL. Chromatography was performed using a mobile phase of 0.3 M ammonium hydrogen carbonate buffer (pH = 8.0–8.1) at a flow rate of 0.5 mL/min. Protein standards with known molecular weights composed of lactalbumin (14,073 Da), r-insulin (5807 Da), and vitamin B12 (1355 Da) were used. The molecular weight of the collected samples could be estimated from a graph of the logarithm of the molecular weight as a function of the retention time of the protein standards.

## 3. Results

### 3.1. Amino Acid Concentrations

**Figure 1:**
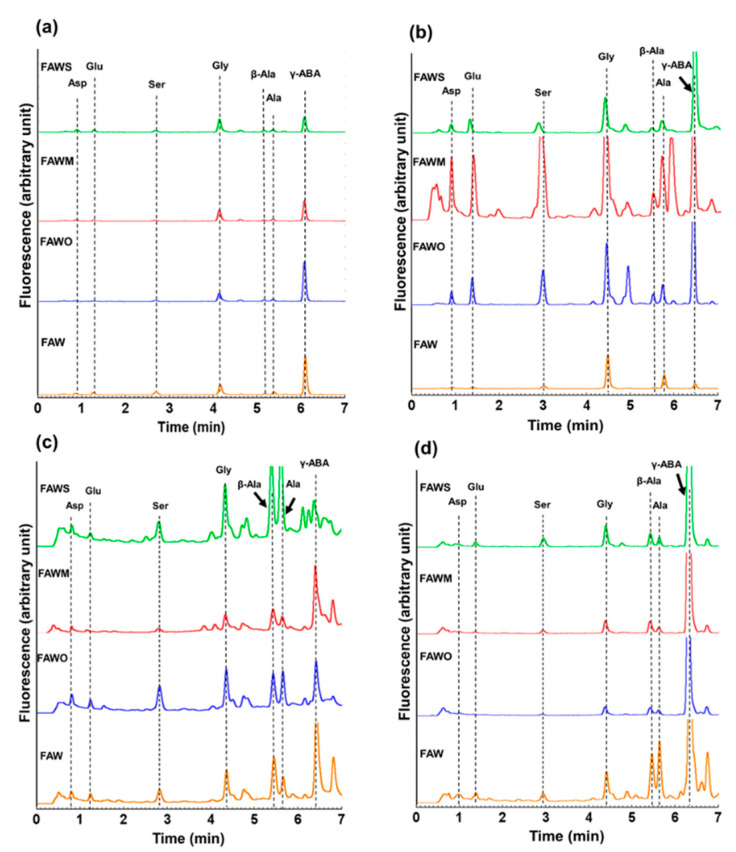
Chromatograms of the acid-hydrolyzed (**a**) unheated formaldehyde-ammonia-water (FAW) treated at RT (0 d), and FAW heated at 150 °C for (**b**) 1 d, (**c**) 3 d, and (**d**) 7 d under the following conditions: without minerals “FAW”, with added olivine “FAWO”, with added montmorillonite “FAWM”, and with added serpentine “FAWS”. Asp: aspartic acid, Glu: glutamic acid, Ser: serine, Gly: glycine, Ala: alanine, β-Ala: β-alanine, γ-ABA: γ-aminobutyric acid.

**Figure 2:**
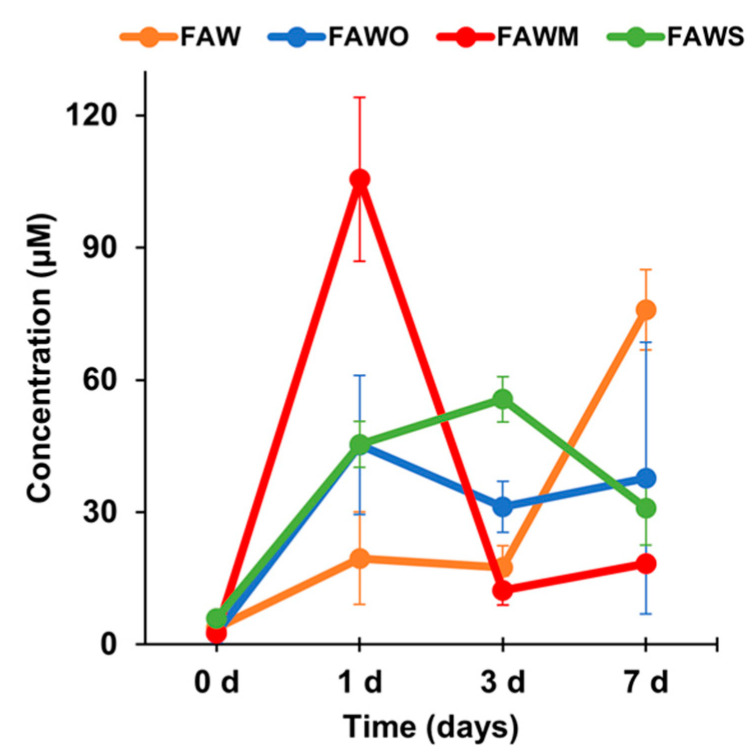
Total amino acid concentrations (control-corrected values) of the unheated FAW samples treated at RT (0 d) and the heated FAW at 150 °C for 1 d, 3 d, and 7 d after acid hydrolysis. Note that the error is the standard deviation (1 σ) of three or more separate experimental runs. The total amino acids include six amino acids: Gly, Ala, β-Ala, Ser, Asp, and Glu.

**Figure 3:**
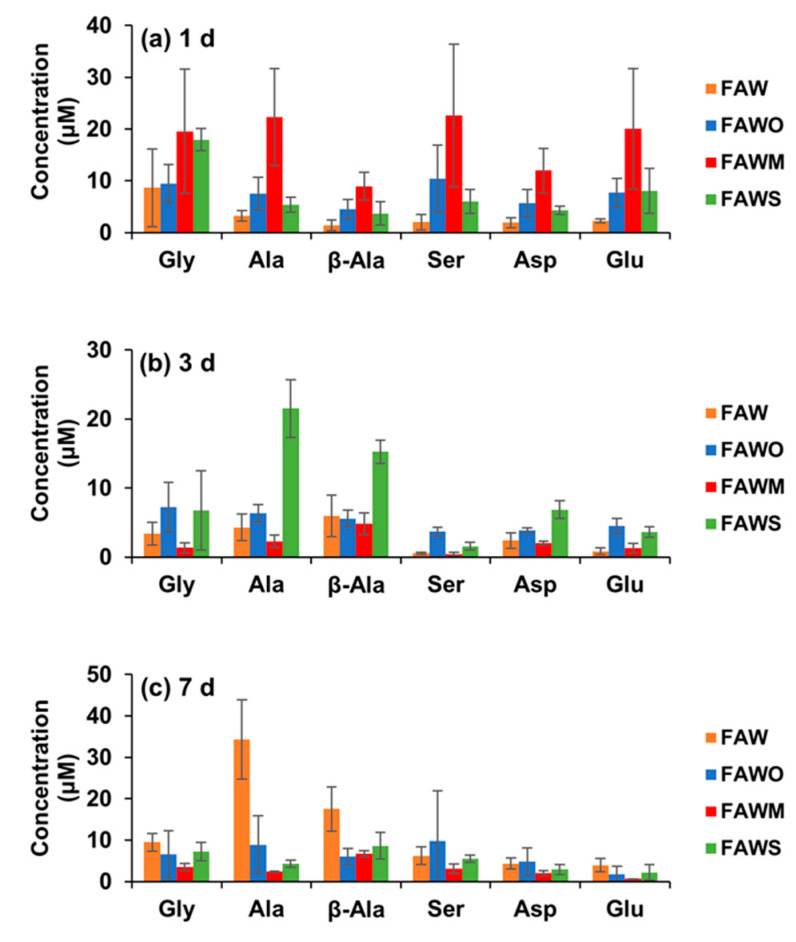
Amino acid concentrations (control-corrected values) in the FAW samples (with and without minerals) heated at 150 °C after acid hydrolysis for (**a**) 1 d, (**b**) 3 d, and (**c**) 7 d. The error is the standard deviation (1 σ) of three or more separate experimental runs.

**Figure 4:**
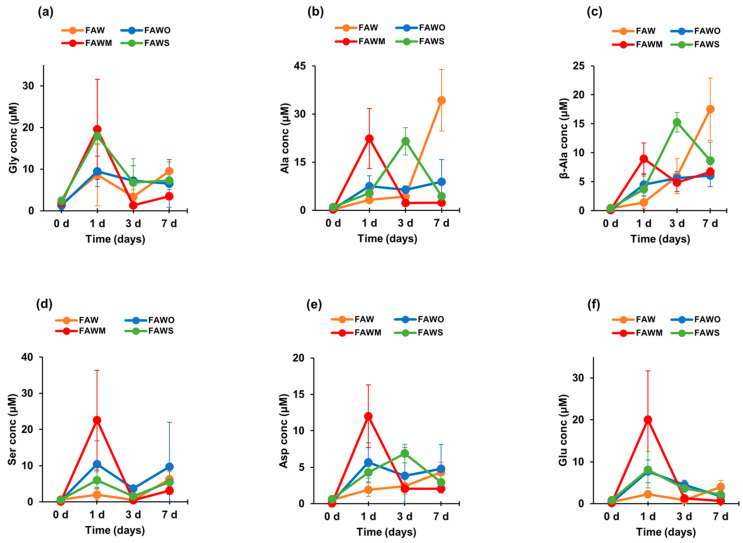
The concentrations (control-corrected values) of (**a**) Gly, (**b**) Ala, (**c**) β-Ala, (**d**) Ser, (**e**) Asp, and (**f**) Glu of the unheated FAW treated at RT (0 d) and the FAW samples heated at 150 °C for 1 d, 3 d, and 7 d after acid hydrolysis. The error is the standard deviation (1 σ) of three or more separate experimental runs.

After acid hydrolysis, the amino acids concentrations of the heated mixture of formaldehyde and ammonia in the presence of water (FAW) with and without minerals were quantified using UHPLC. Figure 1 displays typical chromatograms of the unheated FAW with and without minerals, treated at RT (0 day samples), and the heated FAW with and without minerals, heated at 150 °C for 1 d, 3 d, and 7 d, comparing the addition of different minerals. To understand the relative peak sizes, the y-scale and x-scale used for all chromatograms in Figure 1 are the same. Amino acids were identified by comparing their retention time with those in the known standards (Figure S1). After acid hydrolysis, all FAW mixtures with and without minerals including the unheated FAW treated at RT (0 d) and those heated at different heating periods (1 d, 3 d, and 7 d) exhibited a wide range of amino acids including glycine (Gly), alanine (Ala), β-alanine (β-Ala), serine (Ser), aspartic acid (Asp), glutamic acid (Glu), and γ-aminobutyric acid (γ-ABA). The concentrations (μM) of the amino acids (Table 1, Figure 2, Figure 3 and Figure 4) are calculated from control-corrected values of three or more separate measurements that were obtained by subtraction of the control samples (without ammonia under the same experimental conditions) from the amino acid quantities of the FAW. Although the γ-ABA and some other peaks appeared clearly in some samples, we could not discuss them, since their concentrations showed unexplained errors. For all the unheated FAW (0 d) treated at RT, the total amino acids concentrations were only small amounts with and without minerals. The total amino acids concentrations for FAW without minerals, with olivine, with montmorillonite, and with serpentine were 3.8 ± 2.0, 2.6 ± 2.2, 2.8 ± 0.8, and 5.8 ± 1.1 μM, respectively. Gly was the dominant amino acid in all unheated FAW samples and its concentrations for FAW without minerals, with olivine, with montmorillonite, and with serpentine were 1.6 ± 1.0, 1.3 ± 1.1, 2.0 ± 0.4, and 2.4 ± 0.2 μM, respectively.

Figure 2 presents the change in total amino acid concentrations with time depending on mineral addition after acid hydrolysis. After heating at 150°C, the concentration of the amino acids increased significantly for all samples with and without minerals. For FAW without minerals, the total amino acid concentration after heating for 1 d and 3 d were 19.5 ± 10.5 and 17.4 ± 4.9 μM, respectively, and then increased to 75.9 ± 9.1 μM for 7 d. However, these results changed in response to the addition of minerals, and the concentrations of the amino acids have different response behaviors according to different minerals. For the 1 d experiment, the total amino acids concentrations were almost the same in the presence of olivine and serpentine at around 40 μM. After increasing the heating duration from 1 d to 7 d, the total amino acid yields were almost constant at around 40 μM. Therefore, it seems that olivine and serpentine had almost the same effect on the total amino acid formation. As a consequence, the total amino acids with these minerals were higher than the case without minerals at 1 d and 3 d, yet lower than without minerals at 7 d. Montmorillonite had a different effect on amino acid production, since the amino acids production at 1 d was the highest among all tested samples, whereas the amino acid concentrations decreased sharply after heating for 3 d and 7 d to the lowest observed concentration. Overall, montmorillonite enhanced amino acid production after heating for 1 d, while olivine and serpentine enhanced production for 3 d. After 7 d, all minerals had a negative effect on the production of the amino acids.

Figure 3 and Figure 4 show each amino acid concentration (after acid hydrolysis) in the heated FAW samples. In FAW without minerals, the Gly concentrations for 1–7 d were almost stable with time within the margin of error, despite the slight changes—the concentration of Gly heated for 1 d was 8.7 ± 7.5 μM and became 3.4 ± 1.7 μM, after heating for 3 d. Its concentration increased again to 9.5 ± 2.2 μM after heating for 7 d (Figure 4a). The rest of the amino acids showed a relative increase, especially Ala and β-Ala, which increased notably after heating for 7 d. The concentrations of Ala and β-ala heated for 1 d were 3.3 ± 1.0 μM and 1.4 ± 1.1 μM, respectively, increasing to 4.3 ± 1.9 μM and 5.9 ± 3.0 μM, respectively, after 3 d heating; then the concentrations increased significantly after 7 d heating, increasing to 34.3 ± 9.6 μM and 17.5 ± 5.4 μM, respectively (Figure 4b,c). The dominant amino acid in 1 d heated FAW without minerals was Gly; however, β-Ala and Ala became the most abundant amino acids in the FAW heated for 3 d and 7 d (Figure 3). Our results for the amino acid production from hydrothermal experiments conducted for 3 d without mineral addition revealed that β-Ala was the most abundant amino acid, followed by Ala then Gly, even though their concentrations seemed to be very close. However, the experimental results of Kebukawa et al. [29] (experimental details are provided in the discussion section) from hydrothermal experiments performed at 150 °C for 3 d revealed that Ala was the most dominant, followed by Gly then β-Ala.

After adding olivine, the four amino acids, Gly, Ala, β-Ala, and Asp were produced at 3 d and 7 d with similar amounts to those at 1 d heating, in contrast to Glu, which continuously decreased at 3 d and 7 d (Figure 4). All amino acid concentrations were the highest with montmorillonite among the cases with other minerals and without minerals at 1 d, and α-amino acids were dominant (Figure 3a). Subsequently, all amino acids decreased drastically after 3 d and 7 d (Figure 4), and Ala and β-Ala became the dominant amino acid in some cases at 3 d and 7 d (Figure 3b,c). With serpentine, the dominant amino acid at 1 d was Gly, followed by Ala and β-Ala, which became dominant after 3 d; the concentrations of these amino acids decreased after 7 d and β-Ala and Gly became dominant (Figure 3). Ser and Asp were relatively stable with time (Figure 4d,e), while Gly and Glu decreased gradually with time (Figure 4a,f). It should be noted that Gly decreased with time, but even Gly became dominant at 7 d with the addition of serpentine (all amino acids decreased at 7 d). Ala, β-Ala, and Asp yields were enhanced by serpentine at 3 d, then decreased again after 7 d (Figure 4b,c,e).

### 3.2. FTIR Absorption Spectra

**Figure 5:**
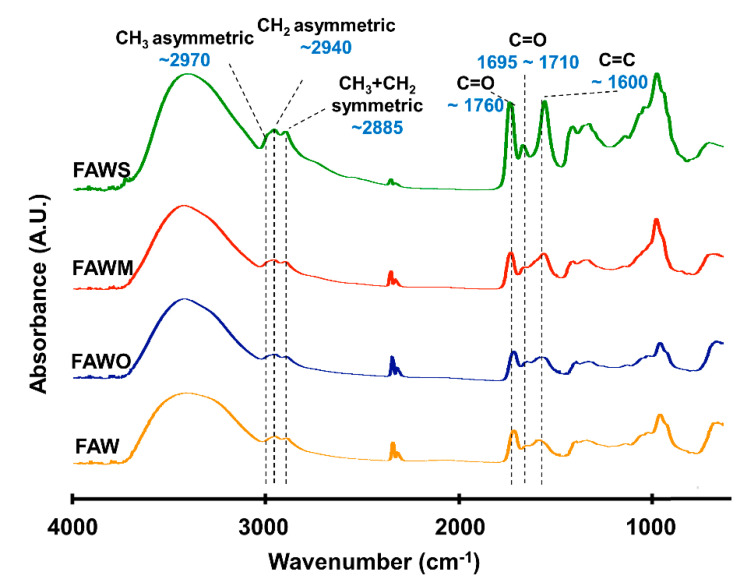
Micro-Fourier transform infrared (FTIR) spectra for the region of 700–4000 cm^−1^ of FAW heated at 150 °C for 1 d.

**Figure 6:**
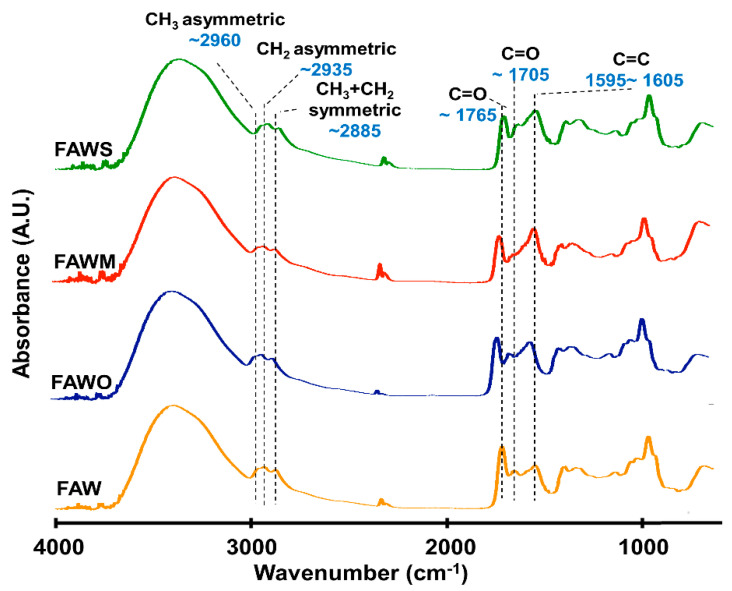
Micro-FTIR spectra for the region of 700–4000 cm^−1^ of FAW heated at 150 °C for 3 d.

**Figure 7:**
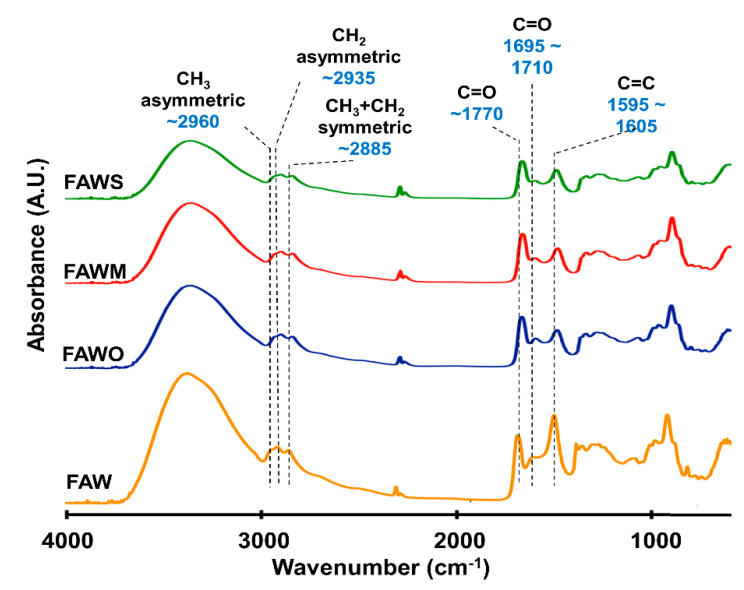
Micro-FTIR spectra for the region of 700–4000 cm^−1^ of FAW heated at 150 °C for 7 d.

FTIR was used to track the changes in the bulk molecular structures of the heated mixtures of formaldehyde and ammonia solutions (FAW) at different heating durations with various mineral additions. Various functional groups were detected in the IR absorption spectra of dried solutions of heated FAW (Figure 5, Figure 6 and Figure 7) and the identified peak assignments are summarized in Table 2. Figures S2–S4 show the IR spectra of the liquid phase (dried) of heated FAW and the sample solutions without ammonia under the same experimental conditions as control for amino acid concentrations. The heated FAW exhibited absorption bands (1) in the region of 3685–3000 cm^−1^, characteristic of OH stretching modes including carboxyl, alcohol, and associated water, (2) at 2960–2970 and 2935–2940 cm^−1^ due to CH_3_ asymmetric stretching and CH_2_ asymmetric stretching, respectively, and at the 2885 cm^−1^ band containing both CH_3_ and CH_2_ symmetric stretching, since the symmetric stretching bands of CH_2_ at 2870–2840 cm^−1^ and CH_3_ at 2885–2865 cm^−1^ cannot be clearly separated, (3) at approximately 1760, approximately 1700, and 1600 cm^−1^ due to C=O (ester), C=O (carboxyl, aldehyde, and amide), and aromatic C=C bands, respectively, and (4) at 1050 cm^−1^ due to C–O stretching modes [39]. It should be noted that no peaks from silicates were observed since mineral phases (solid phases) were separated from the FAW solutions by centrifuging.

**Figure 8:**
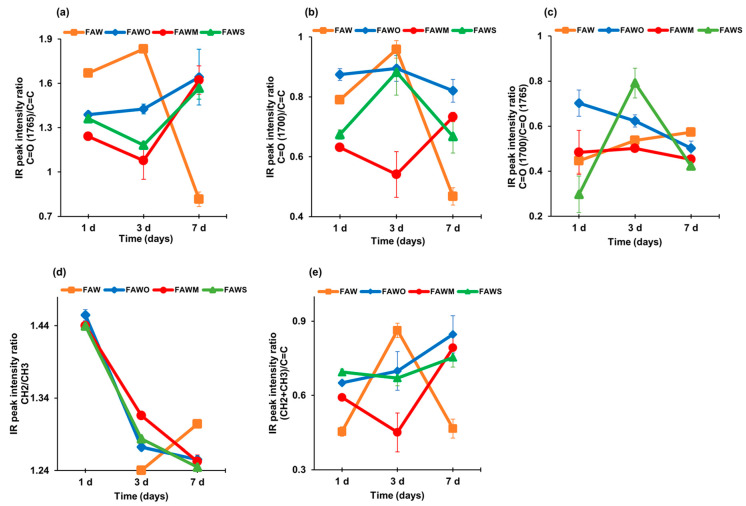
Trends in relative organic functional group concentrations with time for the soluble organics of FAW (with and without minerals) as determined by FTIR for the peak intensity ratios of (**a**) C=O (ester)/C=C, (**b**) C=O (carboxyl, aldehyde, amide)/C=C, (**c**) C=O (carboxyl, aldehyde, amide)/C=O (ester), (**d**) CH_2_/CH_3_, and (**e**) (CH_2_ + CH_3_)/C=C. The C=O (ester), C=O (carboxyl, aldehyde, amide), and C=C peak intensities are obtained by the peak top height of the band around 1765, 1700, and 1600 cm^−1^, respectively, with the linear baseline between 1840 and 1520 cm^−1^. The CH_2_ and CH_3_ peak intensities are obtained by the peak top height of the band at 2935 cm^−1^ (CH_2_) and the band at 2960 cm^−1^ (CH_3_), respectively, with the linear baseline between 3010 and 2790 cm^−1^.

The IR absorption spectra of heated FAW showed a relative change due to mineral addition as well as increasing heating duration. The reproducibility was confirmed, since micro-FTIR spectra were obtained from several different areas for each sample, and the average of the peak height ratios and the standard deviation of the mean (1 σ) was calculated (see Section 3.2.1, Figure 8, and Table S1)—the error could be due to the heterogeneity of the sample. There were some changes in the peak positions of functional groups under the effect of increasing heating durations and adding minerals (Table 3). For instance, aliphatic CH_3_ and CH_2_ peaks were equally shifted to lower wavenumbers, from 2970 cm^−1^ and 2945 cm^−1^, respectively, down to 2960 cm^−1^ and 2935 cm^−1^, respectively, under the effect of time and minerals. The C=O stretch peak shifted from 1760 cm^−1^ (the ester region) to higher wavenumbers (1770 cm^−1^) under the effect of minerals and time. It should be noted that the peak positions of the C=C stretching bands around 1600 cm^−1^ decreased from 1645 cm^−1^ to 1595 cm^−1^ with time in FAW without minerals. In FAW with olivine, the C=C stretch peak position decreased from 1620 cm^−1^ to 1600 cm^−1^, while the addition of phyllosilicates (montmorillonite and serpentine) did not show a significant difference in the peak position with time. The C=C peak position changed notably after adding minerals at 1 d of heating.

#### 3.2.1. IR Peak Intensity Ratios

Since the peaks of C=O at approximately 1765 cm^−1^ and 1700 cm^−1^, and C=C at approximately 1600 cm^−1^ were clearly distinguished (Figure 5, Figure 6 and Figure 7), the peak height ratios were obtained with a linear baseline correction between 1840 and 1520 cm^−1^. Figure 8a shows peak height ratios of ~1765 cm^−1^ (C=O in ester) over ~1600 cm^−1^ (C=C). This ratio exhibited an increase in value with time in the presence of minerals and decreased with time without minerals. Figure 8b shows the peak height ratios of ~1700 cm^−1^ (C=O in carboxyl, aldehyde, and amide) over ~1600 cm^−1^ (C=C). This ratio exhibited a relative variation with time and the addition of minerals, since it decreased with time in FAW samples except in FAW with montmorillonite, and decreased after heating for 3 d, yet finally increased after heating for 7 d. The peak height ratio of ~1700 cm^−1^ (C=O in carboxyl, aldehyde, and amide) and ~1765 cm^−1^ (C=O in ester) were also plotted (Figure 8c). This ratio exhibited a relative decrease with time in the presence of minerals; however, it increased in FAW without minerals.

Figure 8e shows the total aliphatic/aromatic ((CH_2_+CH_3_)/C=C) intensity ratios, with higher values with added minerals than for the heated FAW without minerals at 1 d, and this ratio keeps increasing with time. At the same time, FAW without added minerals showed an increase in this ratio at 3 d, which then essentially decreased at 7 d. CH_2_/CH_3_ intensity ratios were obtained from the peak heights of the aliphatic CH_3_ asymmetric stretching (~2960 cm^−1^) and CH_2_ asymmetric stretching (~2935 cm^−1^) bands (Figure 8d). This ratio decreased with time in FAW with minerals; however, in the case of FAW without mineral addition, the ratio increased (from 3 d to 7 d; CH_2_ could not be separated in case of 1 d). Our results with mineral addition are consistent with those of Kebukawa and Cody [40], showing that the CH_2_/CH_3_ ratios decreased with time in the solid organic products synthesized from formaldehyde, glycolaldehyde, ammonia, and water in the presence of Ca(OH)_2_. It should be noted that the peak height ratio is proportional to the relative amounts of CH_3_ and CH_2_ groups in the heated FAW samples. The aliphatic CH_2_/CH_3_ ratio approximately represents the length of the aliphatic chain length and the levels of branching [41]; the higher ratio indicates longer aliphatic chains, and the lower ratio indicates shorter chain lengths or higher branching levels.

### 3.3. Molecular Weight Estimation

**Figure 9:**
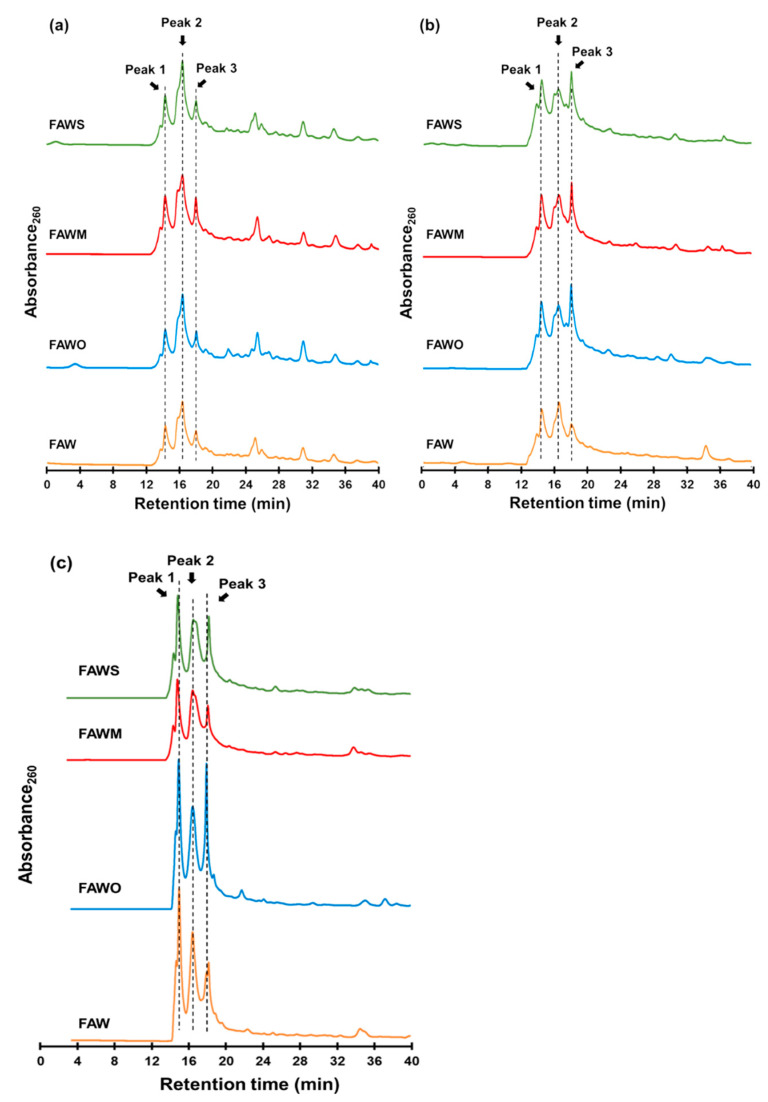
Gel filtration chromatograms of the FAW heated at 150 °C for (**a**) 1 d, (**b**) 3 d, and (**c**) 7 d.

**Figure 10:**
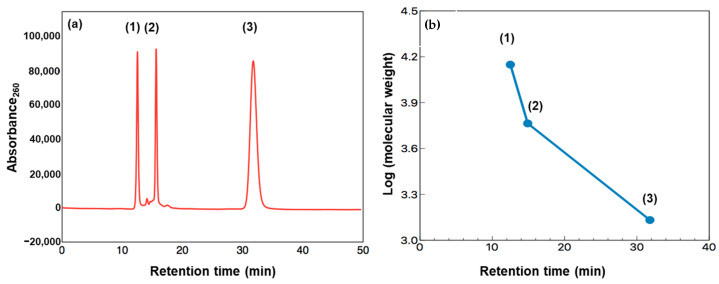
(**a**) Chromatogram of the protein standard: (1) Lactalbumin (Mwt: 14,073 Da, RT: 12.5 min), (2) insulin (Mwt: 5807 Da, RT: 14.9 min), and (3) vitamin B12 (Mwt: 1355 Da, RT: 31.8 min). (**b**) Logarithm of the molecular weight of the protein standard as a function of retention time. Note; Mwt: molecular weight, RT: retention time.

Figure 9 presents gel filtration chromatograms of the heated FAW with and without minerals heated at 150 °C for 1 d, 3 d, and 7 d, respectively. We should note that the original chromatograms of the FAW heated for 7 d were delayed by 3.5 min due to the instrumental conditions, and the delay was corrected. Three major peaks denoted as Peak 1, 2, and 3, could be distinguished in all chromatograms at retention times of 14.4, 16.5, and 18.0 min, respectively. In our experiments, the shape of the detected peaks was approximately the same in all chromatograms. Therefore, we only considered these three major peaks for further interpretation. Peaks eluted at lower retention times corresponded to high molecular weight compounds, while the lower molecular weight compounds eluted at higher retention times. It is suggested that molecules eluted before 20 min have high molecular weights of at least several hundred Da or a few thousand Da. Chromatograms of the protein standard and a logarithm graph of its molecular weight as a function of retention time are also shown in Figure 10. If we assume that FAW behaves like proteins in the gel filtration column, the molecular weights of Peak 1–3 can be estimated as approximately 7930–7380, 5430–5370, and 4990–4960 Da, respectively. However, it should be noted that these molecular weight calculations are uncertain, and it is just an estimation based on the results with the protein molecular weight standards. Thus, our discussion is based on the relative changes in the molecular weight distributions with mineral additions.

**Figure 11:**
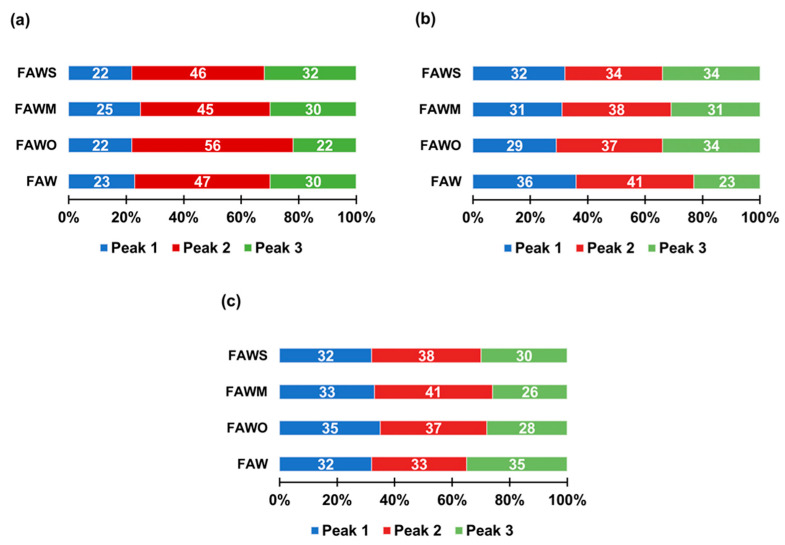
The ratios (%) of each peak area among the total areas of the three major peaks (Peak 1: 12.6–15.2 min, Peak 2: 15.4–17.5 min, and Peak 3: 17.7–20.1 min) of the FAW heated at 150 °C for (**a**) 1 d, (**b**) 3 d, and (**c**) 7 d.

The ratios (%) of each peak area among the total areas of the three major peaks were calculated (Figure 11), (Peak 1: 12.6–15.2 min, Peak 2: 15.4–17.5 min, and Peak 3: 17.7–20.1 min). After heating for 1 d, Peak 2 was dominant compared to Peaks 1 and 3 in FAW with and without minerals. After heating from 1 d to 3 d, the area of Peak 1 (highest molecular weight) increased in FAW both with and without mineral addition. However, the area of Peak 3 (lowest molecular weight) increased with mineral addition, while it decreased without minerals. After heating for 7 d, FAW without minerals showed a decrease in Peak 1 (but still higher than that of 1 d heating), in parallel with an increase of Peak 3. On the contrary, adding minerals showed an increase in the area of Peak 1 with a decrease in the area of Peak 3. These results indicate that increasing heating duration without minerals can enhance the combination of organic molecules. However, the minerals enhanced both the decomposition and combination of organic molecules after 3 d, in addition to their combination after 7 d. Overall, the molecular weights of organic molecules could be affected due to the effect of increasing the heating duration as well as the presence of minerals.

## 4. Discussion

### 4.1. Amino Acid Production during Aqueous Alteration in Small Bodies in the Solar System

Initially, the aqueously altered chondritic parent bodies are composed of silicate-containing icy dust, with the ice consisting of predominantly H_2_O, CO, and CO_2_ with some formaldehyde and ammonia [42]. The internal heating that melted the ice to produce water could be mainly due to the decay of ^26^Al [20,21], with other possibilities such as impact heating [43]. The aqueous alteration conditions have been determined to be mildly alkaline and for CI1 chondrites from 20 °C to 150 °C, for CR chondrites from 50 °C to 150 °C, for CO and CV chondrites from 0 °C to 340 °C, and up to 260 °C for ordinary chondrites [18]. Such conditions are favorable for the formose reaction [44]. Complex macromolecular organic solids were formed by further condensation and carbonization of formaldehyde and glycolaldehyde [27]. Ammonia could enhance the yields of organic solids and amino acid production [28,29,40]. Therefore, in the present study, we used a mixture of formaldehyde and ammonia in the presence of water for simulating asteroids, the remnants of planetesimals. Moreover, we added some minerals to the previous aqueous mixture to study their catalyzing or inhibiting effects on amino acid production and to analyze in-depth the impact of each mineral. The major amino acid products from our hydrothermal experiments after acid hydrolysis were Gly, Ala, β-Ala, Ser, Asp, Glu, and γ-ABA.

The amino acid production from the hydrothermal experiments conducted for 3 d is approximately ten times less than those in the experimental results of Kebukawa et al. [29], when they conducted hydrothermal experiments using formaldehyde, glycolaldehyde, ammonia, and water as a starting solution with a molar ratio of C:N:H_2_O (7.2:0.72:100) in the presence of saturated Ca(OH)_2_. Their results showed that the concentrations of Gly, Ala, and β-Ala heated for 3 d at 150 °C were approximately 300 μM, 500 μM, and 180 μM, respectively. Considering that the C:N:H_2_O ratios are close to those in the present experiments (9:1:100), the presence of glycolaldehyde and Ca(OH)_2_ could enhance the yields of amino acids. However, the high abundance of glycolaldehyde and Ca(OH)_2_ in the meteorite parent bodies are not expected, although glycolaldehyde has been found in comets [45]. Thus, our starting materials are more realistic for the meteorite parent body conditions. Vinogradoff et al. [35] conducted hydrothermal experiments with a starting solution containing hexamethylenetetramine (HMT), and their results showed that, without acid hydrolysis, the concentrations of Gly, Ala, and β-Ala heated for 2 d at 150 °C were 7.7 μM, <0.1 μM, and 1.0 μM, respectively. Their results are consistent with our 1 d experiment results without minerals since Gly was the dominant amino acid in both studies. However, after increasing the experimental duration to 7 d, their results [35] showed that Gly increased significantly to 190.6 μM, still the dominant amino acid, while Ala and β-Ala increased relatively to become 7.0 μM and 7.9 μM, respectively, which is inconsistent with our results in the 7 d experiments, since our Ala and β-Ala increased notably and became dominant.

In the FAW without minerals, the concentration of Gly revealed some stable behavior over time; however, the concentrations of Ala and β-Ala continuously increased with time. The initial formation of the amino acids could be primarily affected by the heating duration, and thus at 1 d, Gly was the dominant amino acid, yet after 3 d and 7 d, Ala and β-Ala showed a significant increase and became dominant. This could be explained primarily by the amino acid decomposition process during thermal alteration of the organic matter via α-decarboxylation, e.g., the decomposition of Asp leading to the formation of β-Ala [46]. Many studies have revealed that α-amino acids are abundant in the Murchison meteorite [46,47,48,49], as well as through hydrothermal synthesis experiments formed from a gas mixture of methane and nitrogen with the principal metal ions present in seawater [50], and a solution containing carbonate, cyanide and formaldehyde [51] and a gas mixture of carbon monoxide, ammonia, and water [9]. In contrast, in the CI1 chondrites Orgueil and Ivuna, which were subjected to an extended aqueous alteration compared to CM and CR chondrites, β-Ala is the most abundant [46,47,48,49]. This is consistent with the relative decrease of Asp with time in our experiments since β-Ala is formed by the decomposition of Asp.

### 4.2. The Effects of Minerals on Amino Acid Formation

Our results from the hydrothermal experiments with the presence of minerals showed that olivine, montmorillonite, and serpentine enhance the amino acid yield at a short heating duration (1 d). Adding montmorillonite to the FAW solution mixture could enhance amino acid production remarkably more than olivine and serpentine after heating for 1 d. Our results are supported by previous studies that demonstrated that montmorillonite enhanced the yield of amino acids in prebiotic conditions simulating early Earth [52,53]. Amino acid preservation is probably promoted by montmorillonite due to its high surface areas and small pore sizes [54].

However, after 3 d and 7 d, the formation of the amino acids decreased significantly with montmorillonite; thus, the decomposition of amino acids could be enhanced by montmorillonite at longer heating durations. Moreover, the production of amino acids was enhanced in the presence of olivine and serpentine at 1 d and 3 d, yet with a longer heating period (7 d), the presence of these minerals decreased amino acid production enhancement. Despite this, while in the presence of olivine, the amino acid concentration decreased after 3 d, it was still higher than for FAW without minerals, and consequently, olivine and serpentine were considered to enhance amino acid production at 1 d and 3 d. After 7 d, all minerals had a negative effect on amino acid production, as they enhanced amino acid decomposition. Overall, it was suggested from our results that minerals enhanced the production of amino acids at short heating durations yet enhanced their decomposition for longer heating durations. It should be noted that our experiments were conducted at relatively high temperature (150 °C) with much shorter duration (days) to enhance the reaction more quickly compared to the parent body process, which proceeded at a lower temperature (possibly close to 0 °C) with much longer duration (millions of years). Such environments cannot be simulated in laboratory experiments, and thus to discuss more realistic and detailed time-temperature dependence of amino acid behavior, kinetics and thermodynamic evaluations would be required.

Meteoritic organic matter is closely correlated with phyllosilicates, proposing that these minerals may have had catalytic effects during molecular evolution in the early solar system [31]. It has been proposed that phyllosilicates have catalytic effects on the synthesis and polymerization of amino acids in the prebiotic Earth environment, e.g., [55,56,57]. The surfaces of phyllosilicates are elementally charged in an aqueous solution, and then amino acids are adsorbed on their surfaces. The adsorption mechanisms of amino acids and phyllosilicates have also been studied [56,58,59,60,61]. The chemical characteristics of specific mineral adsorption sites may affect the determination of amino acid stability. Vinogradoff et al. [34,35] conducted hydrothermal experiments at 150 °C and an alkaline pH, using HMT, in the presence of Al- and Fe-rich phyllosilicates (smectites), finding that the decomposition of HMT to formaldehyde and ammonia leads to the formation of a diverse suite of soluble organic compounds, including amino acids. They found that Fe-rich smectite prohibited the production of amino acids, while the presence of Al-rich smectite stimulated it after 31 days [35]. Thus, the production of the amino acids could be affected by the unique characteristics of smectites, such as structure, chemical composition, and crystal size. In our hydrothermal experiments at 150 °C and an alkaline pH, using formaldehyde and ammonia, the presence of smectite (montmorillonite) induced the enhancement of amino acid production at 1 d and the decomposition of amino acids after longer heating. This could be due to the changes in the structure of montmorillonite during heating.

### 4.3. Molecular Structure Variations of FAW with Minerals

Here we discuss the bulk molecular structures of FAW, which include the amino acid precursors based on the IR spectra of the dried solutions. The ester C=O/C=C peak intensity ratios in FAW without minerals showed a higher value after heating for 1 d and 3 d than these ratios in FAW with minerals. This could be due to the enhancement of the decarboxylation by the minerals at short heating durations; while, after heating for 7 d, the addition of minerals seems to increase this ratio, which may be due to catalytic esterification at longer heating durations. The presence of both Brønsted and Lewis acid sites in phyllosilicates can make them natural esterification catalysts [62]. The sources of Brønsted and Lewis acidity are the hydrated and dehydrated cations, respectively, in the interlayer space and the surface of phyllosilicates [63]. Olivine also contains Lewis acid sites [64], which promote the esterification of FAW.

The peak intensity ratio of CH_2_/CH_3_ is a good index of the length and branching of the aliphatic chain. The CH_2_/CH_3_ peak intensity ratio is not equal to its molar abundance ratio due to the difference in molar absorption coefficients between CH_2_ and CH_3_; however, there is a linear correlation between the CH_2_/CH_3_ peak intensity ratio and the actual number of CH_2_/CH_3_ in molecules [41]. Higher CH_2_/CH_3_ ratios refer to longer aliphatic chains or higher contents of cyclic aliphatic structures, whereas shorter chain lengths or higher branching levels are suggested by lower CH_2_/CH_3_ ratios. The average CH_2_/CH_3_ peak intensity ratios of the FAW with minerals heated for 1 d, 3 d, and 7 d were 1.445 ± 0.005, 1.291 ± 0.003, and 1.251 ± 0.006, respectively. The CH_2_/CH_3_ ratio in heated FAW with minerals was higher than that ratio without minerals for at least the 1 d and 3 d heating durations (Figure 8d), suggesting that the minerals enhance the aliphatic chain length.

Interestingly, The CH_2_/CH_3_ peak intensity ratios of the FAW were consistent with the reported CH_2_/CH_3_ peak intensity ratio of the IOM from the least heated chondrites (1.2–1.4) [65] within analytical error, although our data was from soluble fractions and a large fraction of organic matter in meteorites are insoluble. Therefore, this implies that there is a link between our soluble organic compounds in FAW and that of the IOM, and perhaps the soluble organics condensed into large macromolecules and became IOM-like and/or perhaps some fraction of soluble organics was produced as an alteration product of IOM by parent body processes [66,67].

Adding minerals could affect the nature of C=C since it was suggested that a higher wavenumber indicated a more olefinic nature, and a lower wavenumber indicated a more aromatic nature [39]. The wavenumber of the C=C peaks decreased after adding minerals at 1 d (Table 3), and therefore C=C in FAW without minerals has a more olefinic nature. This then became more aromatic after adding minerals, which might be due to cycloaddition enhancement by the minerals. Phyllosilicates are recognized as effective for redox reactions, including cycloadditions [68], due to the existence of both Brønsted and Lewis acid sites [69]. Besides, increasing the heating duration could also affect the nature of C=C in FAW without minerals and FAW with olivine, since their wavenumber decreased and became more aromatic at longer heating durations; however, FAW with phyllosilicates did not show any difference with heating. The C=O (ester) shifted slightly to a higher wavenumber (1760 cm^−1^ to 1770 cm^−1^) due to the presence of minerals as well as increased heating duration. This slight increase in the wavenumber of the peaks in FAW with minerals and after longer heating refers to an increase in the force constant of that bond, which suggests the enhancement of bond strength [70]; the increase in the force constant of bonding peaks is likely to refer to an increase in the rigidity of bonds in the molecule [71].

High molecular weight organic compounds, estimated at several hundred or a few thousands, were formed in these hydrothermal experiments, which indicated that a solution mixture of formaldehyde and ammonia in the presence of water might be characterized as amino acid precursors that remain stable at longer heating durations, providing various amino acids after acid hydrolysis. The minerals used in this study, olivine and phyllosilicates (montmorillonite and serpentine), might have mostly similar effects and seem to enhance both the decomposition and combination of organic molecules.

## 5. Conclusions

Hydrothermal experiments were conducted using a mixture of formaldehyde and ammonia in the presence of water to simulate aqueous alteration in small bodies in the Solar System. Since the presence of minerals may play significant roles in the formation of organics as well as their evolution in the early Solar System through the catalyzation of various organic reactions, we added three kinds of minerals one by one to the mixture solution of H_2_CO, NH_3_, and water to evaluate their enhancing and inhibiting effects on amino acid production. The FAW produced various kinds of amino acids after acid hydrolysis. Amino acids released after the heating experiments showed different behaviors: Gly remained mostly stable over time while Ala and β-Ala showed a significant increase after 7 d of heating. The minerals enhanced the formation of amino acids at shorter heating durations (1 d), especially with montmorillonite, where the amino acid production was the highest among all tested samples; however, after 3 d and 7 d, the formation of the amino acids decreased significantly with montmorillonite. Olivine and serpentine enhanced amino acid production for 1 d and 3 d heating durations. After 7 d, all minerals had a negative effect on amino acid production, as they enhanced amino acid decomposition. The total concentration of amino acids with olivine and serpentine showed stable behavior with time at around 40 μM for 1 d to 7 d. Accordingly, we suggest that minerals enhance the amino acid production at a short heating duration and enhance their decomposition for longer heating durations. We proposed that the concentrations of the amino acids depend on (1) the presence and the kind of minerals and (2) the stability of the amino acid during longer heating durations. IR spectra of the soluble fraction of FAW (dried) revealed that the ester C=O/C=C peak intensity ratios in the presence of minerals were lower at short heating duration, which might be due to the enhancement of decarboxylation by minerals, and increased after heating for 7 d, which might be due to catalytic esterification by minerals. Gel filtration chromatography indicated that high molecular weight organic compounds were formed from the FAW solution mixture, which started to decompose after a longer heating duration (7 d) without minerals, while the addition of minerals enhanced both the decomposition and combination of organic molecules. Finally, our data showed that minerals affected the formation of amino acids in aqueous environments simulating small bodies in the early Solar System, and the amino acids could have different response behaviors according to the presence of various minerals.

## Figures and Tables

**Table 1:** A summary of the average control-corrected amino acid concentrations in the FAW after acid hydrolysis. FAW: without minerals, FAWO: with olivine, FAWM: with montmorillonite, and FAWS: with serpentine.

Amino Acid Concentration (μM)	FAW				FAWO			
	RT	150 °C			RT	150 °C		
	0 d	1 d	3 d	7 d	0 d	1 d	3 d	7 d
Gly	1.6 ± 1.0	8.7 ± 7.5	3.4 ± 1.7	9.5 ± 2.2	1.3 ± 1.1	9.4 ± 3.7	7.2 ± 3.6	6.6 ± 5.7
Ala	0.3 ± 0.1	3.3 ± 1.0	4.3 ± 1.9	34.3 ± 9.6	0.3 ± 0.2	7.5 ± 3.2	6.4 ± 1.2	8.9 ± 7.0
β-Ala	0.4 ± 0.3	1.4 ± 1.1	5.9 ± 3.0	17.5 ± 5.4	0.1 ± 0.08	4.5 ± 1.9	5.6 ± 1.2	6.0 ± 1.9
Ser	0.6 ± 0.2	2.0 ± 1.5	0.6 ± 0.2	6.2 ± 2.1	0.4 ± 0.3	10.4 ± 6.5	3.7 ± 0.6	9.7 ±12.2
Asp	0.5 ± 0.3	1.9 ± 0.9	2.4 ± 1.1	4.3 ± 1.3	0.24 ± 0.2	5.7 ± 2.7	3.8 ± 0.4	4.8 ± 3.3
Glu	0.4 ± 0.2	2.2 ± 0.4	0.9 ± 0.5	4.0 ± 1.6	0.3 ± 0.2	7.7 ± 2.7	4.5 ± 1.1	1.7 ± 1.9
Total	3.8 ± 2.0	19.5 ± 10.5	17.5 ± 4.9	75.8 ± 9.1	2.6 ± 2.2	45.2 ± 15.8	31.2 ± 5.8	37.7 ± 30.8
**Amino Acid Concentration (μM)**	**FAWM**				**FAWS**			
	**RT**	**150 °C**			**RT**	**150 °C**		
	**0 d**	**1 d**	**3 d**	**7 d**	**0 d**	**1 d**	**3 d**	**7 d**
Gly	2.0 ± 0.4	19.6 ± 12.0	1.3 ± 0.7	3.5 ± 0.9	2.4 ± 0.2	18.0 ± 2.1	6.8 ± 5.8	7.3 ± 2.2
Ala	0.2 ± 0.1	22.4 ± 9.4	2.3 ± 0.9	2.4 ± 0.2	1.0 ± 0.4	5.4 ± 1.4	21.5 ± 4.2	4.3 ± 0.9
β-Ala	0.2 ± 0.1	9.0 ± 2.7	4.8 ± 1.5	6.7 ± 0.7	0.4 ± 0.02	3.7 ± 2.3	15.3 ± 1.7	8.7 ± 3.2
Ser	0.1 ± 0.06	22.6 ± 13.8	0.4 ± 0.3	3.1 ± 1.1	0.6 ± 0.4	6.0 ± 2.3	1.6 ± 0.6	5.5 ± 0.8
Asp	0.1 ± 0.04	12.0 ± 4.3	2.0 ± 0.3	2.0 ± 0.6	0.6 ± 0.04	4.3 ± 0.8	6.9 ± 1.3	3.0 ± 1.2
Glu	0.2 ± 0.1	20.0 ± 11.6	1.3 ± 0.7	0.6 ± 0.03	0.8 ± 0.1	8.1 ± 4.3	3.6 ± 0.8	2.2 ± 1.9
Total	2.8 ± 0.8	105.6 ± 18.6	12.1 ± 3.3	18.3 ± 1.6	5.8 ± 1.1	45.5 ± 5.2	55.7 ± 5.1	31.0 ± 8.5

**Table 2:** Summary of IR peak assignments of FAW based on Socrates [39].

Peak Position (Wavenumber/cm^−1^)	Assignments	Species
3370	OH	Carbonyl, alcohol, associated water
2960–2970	C–H asymmetric stretch	Aliphatic CH_3_
2935–2940	C–H asymmetric stretch	Aliphatic CH_2_
2885	C–H symmetric stretch	Aliphatic CH_3_ + CH_2_
1760–1770	C=O stretch	Ester
1695–1710	C=O stretch	Carboxyl, aldehyde, amide
1595–1605	C=C stretch	Olefinic, aromatic
1050	C–O stretch	

**Table 3:** Summary of functional group peak positions of the FAW.

Peak Position (Wavenumber/cm^−1^)	FAW			FAWO			FAWM			FAWS		
	1 d	3 d	7 d	1 d	3 d	7 d	1 d	3 d	7 d	1 d	3 d	7 d
**CH_3_**	- *	2960	2960	2970	2960	2960	2970	2960	2960	2970	2960	2960
**CH_2_**	2945	2938	2935	2940	2935	2935	2940	2940	2935	2935	2935	2935
**C=O (ester)**	1760	1765	1770	1760	1765	1770	1765	1768	1770	1770	1665	1770
**C=O (Carboxyl, aldehyde, amide)**	1700	1705	1695	1698	1705	1705	1695	1700	1710	1710	1695	1710
**C=C**	1645	1603	1595	1620	1603	1600	1600	1595	1600	1600	1605	1605

* CH_3_ peak could not be separated for FAW 1 d.

## Data Availability

The datasets in this study are available from the corresponding author on reasonable request.

## Supplementary Materials

The following are available online at https://www.mdpi.com/2075-1729/11/1/32/s1, Figure S1: Chromatograms of (a) the amino acid standard, and the acid-hydrolyzed samples with and without ammonia (control) heated at 150 °C for 1 d under the following conditions: (b) without minerals “FAW”, (c) with added olivine “FAWO”, (d) with added montmorillonite “FAWM”, and (e) with added serpentine “FAWS”, Figure S2: Micro-FTIR spectra for the region of 700–4000 cm^−1^ of the solution (with and without ammonia) heated at 150 °C for 1 d under the following conditions: (a) without minerals, (b) with olivine, (c) with montmorillonite, and (d) with serpentine, Figure S3: Micro-FTIR spectra for the region of 700–4000 cm^−1^ of the solution (with and without ammonia) heated at 150 °C for 3 d under the following conditions: (a) without minerals, (b) with olivine, (c) with montmorillonite, and (d) with serpentine, Figure S4: Micro-FTIR spectra for the region of 700–4000 cm^−1^ of the solution (with and without ammonia) heated at 150 °C for 7 d under the following conditions: (a) without minerals, (b) with olivine, (c) with montmorillonite, and (d) with serpentine, Table S1: IR peak intensity ratios.
