# Supplementary material for: Synthesis of Organic Matter in Aqueous Environments Simulating Small Bodies in the Solar System and the Effects of Minerals on Amino Acid Formation
Source: Life (Basel). 2021 Jan 6;11(1):32. doi: 10.3390/life11010032 (PMC7825434; doi:10.3390/life11010032)
Supplement: Supplementary file 1 [file life-11-00032-s001.pdf]

life

Supplementary Materials of Synthesis of organic matter in aqueous environments simulating small bodies in the Solar System and the effects of minerals on amino acid formation

Amino acid analysis

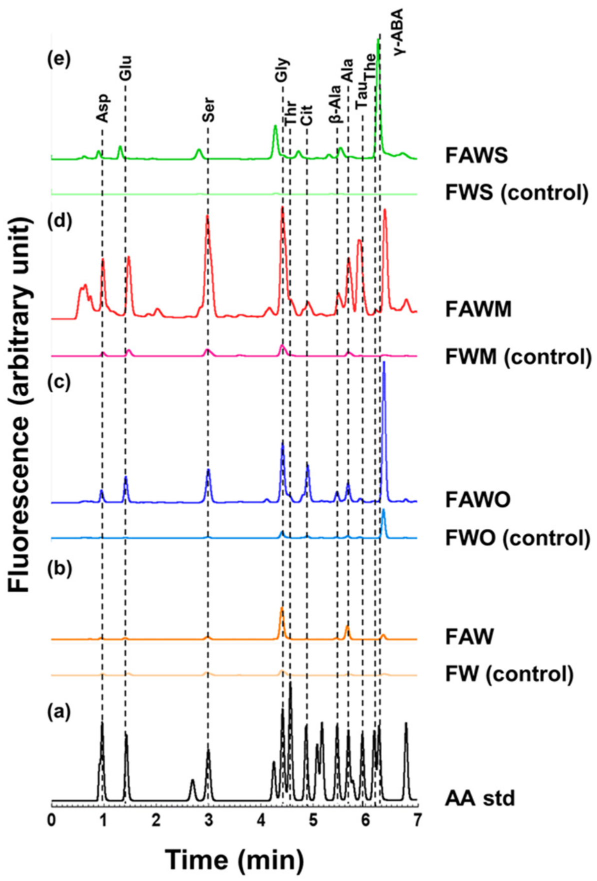

Figure S1. Chromatograms of (**a**) the amino acid standard, and the acid-hydrolyzed samples with and without ammonia (control) heated at 150 °C for 1 d under the following conditions: (**b**) without minerals "FAW", (**c**) with added olivine "FAWO", (**d**) with added montmorillonite "FAWM", and (**e**) with added serpentine "FAWS". Asp: aspartic acid, Glu: glutamic acid, Ser: serine, Gly: glycine, Thr: threonine, Cit: citruline, β-Ala: β-alanine, Ala: alanine, Tau: taurine, The: theanine, γ-ABA: γ-aminobutyric acid.

FTIR absorption spectra without ammonia

Figures 2 to 4 show the IR spectra of the liquid phase (dried) of the heated sample solutions (with and without ammonia) under the same experimental conditions (as control for amino acid concentrations). There were some differences in the functional groups between the IR spectra with and without ammonia. For example, peaks at around 1770 cm^{-1} due to the C=O functional group in FAW without ammonia were not detected. Additionally, the peak heights of aliphatic C-H were markedly higher without ammonia than with ammonia.

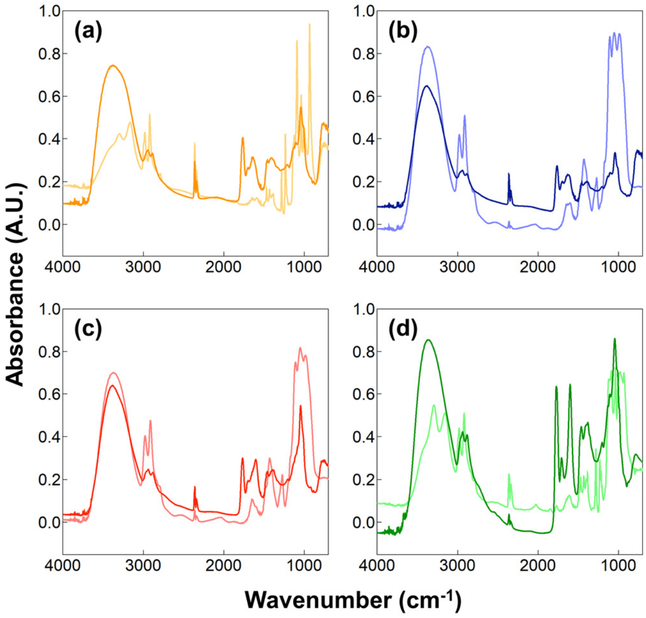

Figure S2. Micro-FTIR spectra for the region of 700–4000 cm^{-1} of the solution (with and without ammonia) heated at 150 °C for 1 d under the following conditions: (**a**) without minerals, (**b**) with olivine, (**c**) with montmorillonite, and (**d**) with serpentine. The faint line is without ammonia.

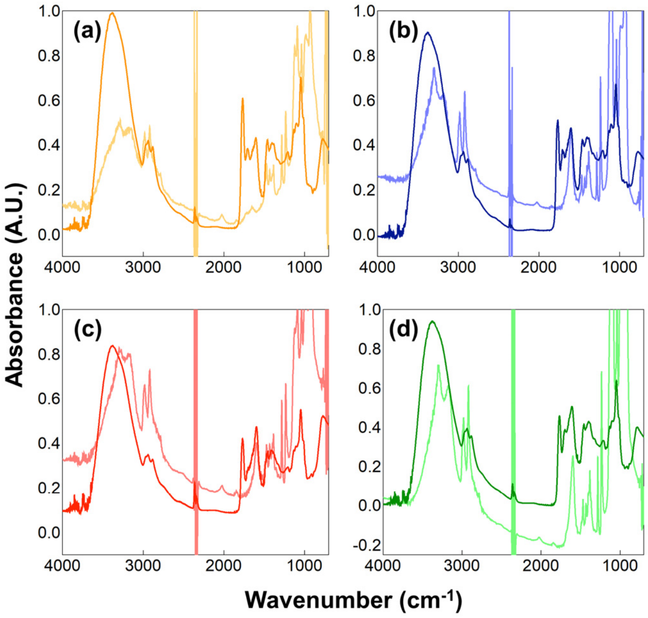

Figure S3. Micro-FTIR spectra for the region of 700–4000 cm^{-1} of the solution (with and without ammonia) heated at 150 °C for 3 d under the following conditions: (**a**) without minerals, (**b**) with olivine, (**c**) with montmorillonite, and (**d**) with serpentine. The faint line is without ammonia.

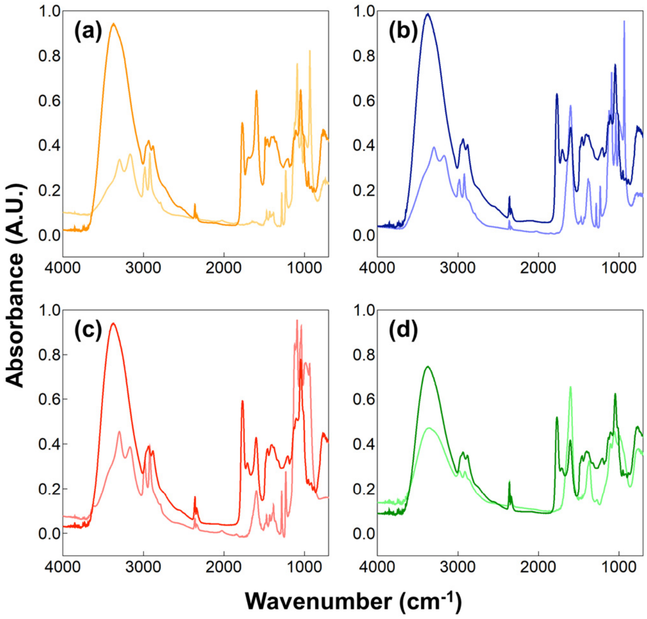

Figure S4. Micro-FTIR spectra for the region of 700–4000 cm^{-1} of the solution (with and without ammonia) heated at 150 °C for 7 d under the following conditions: (**a**) without minerals, (**b**) with olivine, (**c**) with montmorillonite, and (**d**) with serpentine. The faint line is without ammonia.

IR peak intensity ratios

Table S1. IR peak intensity ratios. C=O and C=C peak intensities are obtained by the peak top height of the band around 1765 (ester), 1700 (carboxyl, aldehyde, amide), and 1600 cm^{-1} (C=C), respectively, with the linear baseline between 1840 and 1520 cm^{-1}. The CH_2 and CH_3 peak intensities are obtained by the peak top height of the band at 2935 cm^{-1} (CH_2) and the band at 2960 cm^{-1} (CH_3), respectively, with the linear baseline between 3010 and 2790 cm^{-1}.

C=O (1765)/ C=C	1 d	3 d	7 d	C=O (1700)/ C=C	1 d	3 d	7 d
**FAW**	1.67 ± 0.02	1.83 ± 0.01	0.82 ± 0.05	**FAW**	0.79 ± 0.003	0.96 ± 0.03	0.47 ± 0.03
**FAWO**	1.39 ± 0.01	1.43 ± 0.03	1.64 ± 0.19	**FAWO**	0.87 ± 0.02	0.89 ± 0.04	0.82 ± 0.04
**FAWM**	1.24 ± 0.01	1.08 ± 0.13	1.62 ± 0.10	**FAWM**	0.63 ± 0.01	0.54 ± 0.08	0.73 ± 0.02
**FAWS**	1.36 ± 0.02	1.18 ± 0.02	1.57 ± 0.07	**FAWS**	0.67 ± 0.02	0.88 ± 0.08	0.67 ± 0.01
**C=O (1700)/ C=O (1765)**	**1 d**	**3 d**	**7 d**	**CH_2/CH_3**	**1 d**	**3 d**	**7 d**
**FAW**	0.45 ± 0.06	0.54 ± 0.02	0.57 ± 0.01	**FAW**		1.24 ± 0.002	1.30 ± 0.004
**FAWO**	0.70 ± 0.06	0.62 ± 0.03	0.50 ± 0.03	**FAWO**	1.45 ± 0.01	1.27 ± 0.003	1.26 ± 0.01
**FAWM**	0.48 ± 0.10	0.50 ± 0.01	0.45 ± 0.01	**FAWM**	1.44 ± 0.01	1.32 ± 0.003	1.25 ± 0.01
**FAWS**	0.30 ± 0.08	0.79 ± 0.07	0.42 ± 0.01	**FAWS**	1.44 ± 0.002	1.28 ± 0.002	1.24 ± 0.003
**(CH_2+CH_3)/ C=C**	**1 d**	**3 d**	**7 d**				

**FAW**	0.45 ± 0.02	0.86 ± 0.03	0.47 ± 0.04
**FAWO**	0.65 ± 0.01	0.70 ± 0.08	0.85 ± 0.07
**FAWM**	0.59 ± 0.002	0.45 ± 0.08	0.79 ± 0.05
**FAWS**	0.69 ± 0.01	0.67 ± 0.03	0.75 ± 0.04
